# Emojis in public health and how they might be used for hand hygiene and infection prevention and control

**DOI:** 10.1186/s13756-020-0692-2

**Published:** 2020-02-10

**Authors:** Nasim Lotfinejad, Reza Assadi, Mohammad Hassan Aelami, Didier Pittet

**Affiliations:** 1 Department of Research, Faculty of Medicine, Mashhad University of Medical Sciences, Mashhad, Iran; 2 Hand Hygiene and Infection Control Research Center, Imam Reza Hospital, Mashhad, Iran; 3 E-Learning Center, School of Medicine, Mashhad University of Medical Sciences, Mashhad, Iran; 4 Department of Pediatrics & Hand Hygiene and Infection Control Research Center, Imam Reza Hospital, Mashhad, Iran; 5 Infection Control Programme and WHO Collaborating Centre on Patient Safety, University of Geneva Hospitals and Faculty of Medicine, 4 Rue Gabrielle-Perret-Gentil, 1211 Geneva 14, Switzerland

**Keywords:** Infection prevention and control, Infection control, Emoji, Emoticon, Ideograms, Infection prevention, Hand hygiene, World Health Organization, Healthcare-associated infection, Global health, Behavioral change, Alcohol-based handrub, Hand sanitizer

## Abstract

Emojis are frequently used picture characters known as possible surrogates for non-verbal aspects of behavior. Considering the ability of emojis to enhance and facilitate communication, there has been a growing interest in studying their effects in scientific and health-related topics over the past few years. Infection prevention and control (IPC) is a field of medicine that is directly associated with specific behaviors. These include hand hygiene, which is the cornerstone of the prevention of healthcare-associated infections, and essential in stemming the spread of antimicrobial resistance. This paper aims to provide an overview of how emojis have been used in the medical and public health literature and proposes their possible use in IPC and hand hygiene to put forth a vision for the future research.

## Introduction

Emojis are new generation of emoticons. These ideograms have evolved beyond facial expressions, and are increasingly used on digital platforms to demonstrate concepts and ideas. As a Japanese word meaning “picture character”, emojis were initially created at the end of the twentieth century in order to improve and simplify digital messages [1, 2]. They are increasingly used as a new language worldwide, conveying nonverbal communication cues and, in the case of the latter, substituting for the face-to-face conditions, and proving to exert a direct effect on readers’ moods [3, 4]. Emojis have enabled users from different countries to communicate in a standardized way with single compact characters that circumvent language barriers [5, 6]. The number of existing emojis is increasing each year in a number of categories including smileys and people, animals and nature, food and drinks, activity, travel and places, objects, symbols, and flags [1]. These pictographs became so popular that the Oxford dictionary announced the “face with tears of joy” () emoji as the 2015 word of the year [7]. More than 90% of the online users employ emojis to communicate complex concepts more effectively while using less words [2].

As technology improves and emojis continue to grow in popularity in the digital world, various studies have been conducted in the past few years regarding the impact of these symbols in scientific fields. However, the use of emojis has not yet been highlighted in the medical literature. These surrogates of nonverbal communication have a direct impact on different social interactions that could facilitate communication among healthcare providers and receivers, thus enhancing public health [8]. This paper primarily focuses on the possible effects of emojis on healthcare systems and outlines important issues for further consideration. The potential roles of these symbols in infection prevention and control (IPC), as a globally recognized essential part of health systems [9], are further described through the example of using emojis to promote hand hygiene.

### Emojis in healthcare literature

The ever-evolving medical language, which is globally accepted as medical English, has faced significant changes over time [10]. But despite all the developments that have taken place, language barriers still hinder disseminating knowledge worldwide [11] by preventing sufficient quality of care among patients and healthcare providers who speak different languages and come from different cultures [12, 13]. Numerous pictographs have been developed to enrich verbal communication and facilitate digital communication by substituting for nonverbal cues [8]. They received so much attention that in 2005, a number of medical emoticons were recommended instead of abbreviations and acronyms used in medicine [14]. Emojis are a step further than emoticons. For example, “:-O” is an emoticon for surprise while “” is the emoji with the same meaning. More natural in design, emojis have attracted scientific attention as they are able to transmit emotion, attitude and attention when added to text [1, 15]. As emojis are easily distributed by users around the world, they enable researchers to conduct studies and surveys across geographical boundaries using this language [6].

Shah et al. [5] suggested benefiting from emojis by adding them in editorial communications and writing manuscripts using emojis as substitutes for words to augment medical literature. However, some disadvantages of using emojis in scientific studies were reported such as lack of standardization in different platforms, variability of meaning over time and according to different cultures, and conveyance of unintended messages. Concerning the infiltration of emojis into scientific literature, it is imperative to evaluate the impact of these symbols in health-related fields in order to harness their potential advantages for appropriate research applications and stave off scientific miscommunication. The impact of narrative health messages and emojis on message processing and attention has been studied by Willoughby and Liu [16]. Their findings demonstrated that using emojis in health messages is reliant on the objectives and content of the message. According to the authors, participants’ attention was better when emojis were added to weaker and less interesting health messages. Another study conducted by Siegel et al. [17] on 297 elementary school children revealed that placing “Green Smiley Face” emoticons near plain milk and vegetables increased their desire to purchase healthy products. Lee et al. [18] used emojis as simple, language-independent, and less time consuming tools for detecting depression after stroke. Based on their results, the sad emoticon showed a high agreement rate with the Diagnostic and Statistical Manual for Major Depression, fourth edition, and the use of emojis was found effective in screening for depression among patients. Marengo et al. [19] developed a 91-item questionnaire with emojis related to personality characteristics in order to assess a language-free instrument for personality. The authors involved a sample of 234 young adults online, and a brief Big-Five personality questionnaire was administered along with the emoji questionnaire to each participant. It was concluded that 36 out of 91 emojis were significantly associated with extraversion, emotional stability and agreeableness. Other studies have been conducted regarding the use of emojis, especially in psychology [20–23]; yet there is only limited data on the effects of these pictographs in other fields of medicine. The latter is mainly due to the availability of many emojis describing emotion and behavior, while only a few emojis are precisely related to health and medical terms.

With the dramatic increase in public awareness about the significant role of the Internet and social media in disseminating health-related data [24], Internet access to medical information has gone far beyond reading sources as we are witnessing more interactive communicational methods being introduced each day [25]. Medical emojis have a great potential to gain popularity on digital platforms as the Internet is an integral part of both healthcare workers’ and patients’ lives. Currently, attempts are being made to create new emojis related to health issues, such as the medical sign and symptoms [26]. In a recent study, Assadi et al. have designed and evaluated the possibility of using emojis to depict about 80 clinical signs and symptoms according to the 10th revision of the International Statistical Classification of Diseases [27].

### Emojis in infection prevention and control

Implementing IPC programs is recognized as a global health priority, and it is known that failure to achieve an adequate level of IPC harms patients around the world. That points to the necessity of using a full arsenal of tools and technologies to improve IPC interventions globally [28]. In recent times, trends in infectious diseases have become detectable in populations via the Internet and social media; they function as new and available sources of health-related data [29, 30]. This ability of the Internet and social media was further illustrated in a study of distribution of emojis used on Twitter around the world [31]. The aforementioned study indicated that South-Eastern Asia and South America had the highest, while the USA and Japan had the lowest rates of using emojis on Twitter. It has also been possible to detect the emojis used most often by various countries; based on the results of this study, developed countries used less face emojis than developing countries. It was further concluded that emojis could reflect the living conditions of different counties around the world using correlation analyses between emoji distribution and world development indicators including life expectancy, tax rate, trade, and gross domestic product per capita. According to another study by Kim et al. [32], policy makers should benefit from online information including emoticons along with proven scientific data, since these symbols can represent public feelings like frustration about an infectious disease such as H1N1. Emoticons and Internet slang were also targeted in the process of extracting emotional contents from social media regarding public reaction to the outbreak of Middle East respiratory syndrome in Korea in 2015 [33]. Pointing out the impact of an infectious disease outbreak on public emotions, it was suggested that understanding interactions between a disease, mass media and public emotions might be effective to avoid from excessive fear and overreactions to infectious diseases. Conceivably, by using digital surveillance with the Internet, disease outbreaks could be detected earlier in every part of the world, providing an opportunity to react more quickly [34, 35].

A number of emojis have been created that could be related to infectious diseases. In 2017, the “mosquito” () emoji was proposed with the objective to improve communication and research regarding mosquito-borne diseases and to monitor mosquito-borne disease outbreaks [36]. “Face with thermometer” () and “sneezing face” () are among the smileys that may be used to represent a hospital patient, person with a cold or flu or other physical diseases. New health-related emojis in other categories including “lab coat” (), “microbe” (), “test tube” (), “petri dish” () and “DNA” () have also been added as part of Unicode 11.0 in 2018 to facilitate scientific communication in the digital world.

Considering the significant role of person-to-person transmission of infectious diseases and the necessity of behavioral adherence to IPC interventions, behavioral science is directly associated with infectious disease models [37]. Therefore, the existing set of emojis may be useful for showing behaviors associated with IPC such as hand hygiene. Bearing in mind the limited number of health-related emojis, positive effects of the currently available symbols in the field of IPC are questionable.

### The example of hand hygiene

Healthcare-associated infections are considered a threat to patient safety [38]. Hand hygiene with alcohol-based hand rub, which is the global standard of care, is recognized as the cornerstone of IPC for preventing healthcare-associated infections [39]. Although hand hygiene is a very simple procedure, adherence levels are still worrisome and improving this behavior has been challenging despite using numerous interventions [40, 41]. The core components of the WHO multimodal hand hygiene improvement strategy include system change, education, evaluation and feedback, reminders in the workplace, and institutional safety climate [42]. In order to overcome behavioral barriers multiple interventions have been performed. Emojis may be beneficial in bridging the large gap that exists between the verbal text-based and nonverbal face-to-face interactions related to hand hygiene and the multimodal promotion approach. Thereby, employing them to improve hand hygiene behavior in accordance with the multimodal strategy deserves much more attention.

Education entertainment provided by social media is a promising method to promote individual behavior change such as hand hygiene [43], and emojis are helpful tools to add topics and ideas by facilitating a more natural communication [6]. According to the literature, visual stimuli have been found effective to use in lectures and written texts in order to improve hand hygiene behavior and it has also been suggested that animated visual elements may have a better and more direct effect compared with static pictures [44–46]. Likewise, emotional events are memorized more precisely and for a longer duration compared with neutral events, making them beneficial aspects to consider in education and memory [47]. In line with the possible advantages mentioned for these symbols, the first pilot intervention to assess the impact of emoticons on hand hygiene was performed by Gaube et al. in a German hospital [48]. Monitoring and feedback devices were installed above handrub dispensers that displayed a frowny face to remind people to perform hand hygiene and, once used, a smiley face was shown to reinforce the positive behavior. Based on their findings, emoticons can enhance hand hygiene in hospitals by providing visual cues and preventing forgetfulness concerning when it needs to be performed. Furthermore, emoticons could reinforce the hand hygiene behavior by providing instant feedback in patients’ rooms.

Other studies have pointed to the importance of improving the use of social media platforms by IPC professionals in order to leverage the latest technologies in conveying hand hygiene messages [43, 49, 50]. This was further illustrated in a study conducted in China, which suggested that hand hygiene promotion strategies could be assessed using information obtained from social media, augmenting the data provided by traditional sources such as radio, print, and television [51]. According to the study performed by Pan et al. [50], a hand hygiene campaign video was more effectively connected through Facebook than through a group email and hospital website in Taiwan. It was also recommended that utilization of social media improves hand hygiene programs by providing a safe environment that enables public awareness and includes patient participation. The aforementioned results are consistent with previously performed studies describing the emotional content that is available on Facebook, Twitter, and other social media sites as “contagious” [52, 53]. Therefore, health-related interventions could also be influenced by the emotional cascade effect from one person to others, leading to improved efficacy and increased cost-effectiveness of medical interventions [53]. A preponderance of evidence suggests that it seems beneficial to study the impact of emojis as an inseparable language of the digital platform on the emotional contagion and subsequent behavioral changes regarding hand hygiene.

A wide variety of other new strategies for the use of emojis could be suggested to improve hand hygiene. A qualitative study was performed in Australia to evaluate hand hygiene compliance among hospital cleaners [54]. Despite being aware of the necessity of hand hygiene, adherence levels were still very low in this group. Their results indicated that hand hygiene information overload and confusing training material and programs were barriers to hand hygiene promotion. Using simplified reminders such as posters with comprehensive language may be more beneficial and less time consuming compared with the detailed materials used for training hospital cleaning staff. Another advantage of using emojis in hand hygiene could be the possibility of introducing universal emoji translation of the WHO “My five moments for hand hygiene” [55, 56] with precisely selected emojis in order to prevent misinterpretations by different individuals.

### Current state and future prospects

**Fig. 1:**
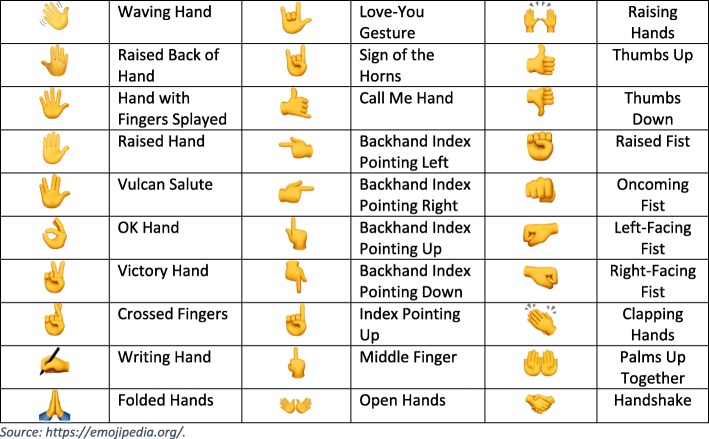
“The existing hand emojis according to unicode 12.0”

Hand hygiene-related terms have been transformed over the past years from handwashing and hand disinfection to hand hygiene, underlining the increased performance and use of alcohol-based handrub compared to soap and water, and the prominence of this topic in patient safety [56, 57]. There are currently 30 different hand gesture emojis available, however there is no emoji directly showing hand washing, handrubbing or handrub (Fig. 1). The act of cleaning hands could be only demonstrated using a sequence of any of the existing hand emojis with the “bar of soap” () emoji, which makes communication more complicated than using a single emoji. For example, some people may interpret the combination of the “clapping hands” and “bar of soap” () emojis as applauding the use of soap and it may not directly demonstrate hand washing with soap. The development of hand hygiene-related emojis will enable health professionals to communicate more specifically regarding their discoveries and concerns in this field. In addition, the hand hygiene compliance of different places in the world could be better notified by detecting relevant emojis in the social media to predict compliance or even identify problems caused by low adherence. Hand hygiene-related emojis on social media could regularly remind us to protect ourselves against healthcare-associated infections, and the spread of influenza or antimicrobial resistance. More importantly, the general public, including patients suffering from healthcare-associated infections, might be able to easily share and communicate the problems they have faced, as they could be better heard globally and have the possibility of sharing their feelings using different emojis.

## Conclusion

Emojis may empower IPC in different aspects such as raising awareness with no language barrier, educating people to adopt healthy behaviors, and enhancing surveillance systems to monitor infectious diseases. It is recommended to evaluate the relevance and appropriateness of the current set of emojis to use for hand hygiene promotion in order to harness the potential beneficial impact of these symbols. Medical emojis that are standardized as a new category of emojis offer much hope for the future, especially in the field of IPC.

## Data Availability

Data sharing not applicable to this article as no datasets were generated or analyzed during the current study.
